# Supplementary material for: Early coauthorship with top scientists predicts success in academic careers
Source: Nat Commun. 2019 Nov 15;10:5170. doi: 10.1038/s41467-019-13130-4 (PMC6858367; doi:10.1038/s41467-019-13130-4)
Supplement: Supplementary file 1 — Supplementary Information [file 41467_2019_13130_MOESM1_ESM.pdf]

Supplementary Information
Early coauthorship with top scientists predicts success in academic careers

Weihua Li,[1,2] Tomaso Aste,[1,2] Fabio Caccioli,[1,2] Giacomo Livan[1,2,*]

[1] *Department of Computer Science, University College London, London WC1E 6EA, United Kingdom*
[2] *Systemic Risk Centre, London School of Economics and Political Sciences, London WC2A 2AE, United Kingdom*

Supplementary Tables

Supplementary Table 1. **Journals in Chemistry (published by American Chemical Society).**

ACCOUNTS OF CHEMICAL RESEARCH
ACS APPLIED MATERIALS & INTERFACES
ACS BIOMATERIALS SCIENCE & ENGINEERING
ACS CATALYSIS
ACS CHEMICAL BIOLOGY
ACS CHEMICAL NEUROSCIENCE
ACS COMBINATORIAL SCIENCE
ACS INFECTIOUS DISEASES
ACS MACRO LETTERS
ACS MEDICINAL CHEMISTRY LETTERS
ACS NANO
ACS PHOTONICS
ACS SUSTAINABLE CHEMISTRY & ENGINEERING
ACS SYNTHETIC BIOLOGY
ANALYTICAL CHEMISTRY
BIOCHEMISTRY
BIOCONJUGATE CHEMISTRY
BIOMACROMOLECULES
BIOTECHNOLOGY PROGRESS

CHEMICAL RESEARCH IN TOXICOLOGY
CHEMISTRY OF MATERIALS
CRYSTAL GROWTH & DESIGN
ENERGY & FUELS
ENVIRONMENTAL SCIENCE & TECHNOLOGY
ENVIRONMENTAL SCIENCE & TECHNOLOGY LETTERS
INDUSTRIAL & ENGINEERING CHEMISTRY RESEARCH
INORGANIC CHEMISTRY
JOURNAL OF AGRICULTURAL AND FOOD CHEMISTRY
JOURNAL OF CHEMICAL EDUCATION
JOURNAL OF CHEMICAL INFORMATION AND MODELING
JOURNAL OF CHEMICAL THEORY AND COMPUTATION
JOURNAL OF MEDICINAL CHEMISTRY
JOURNAL OF NATURAL PRODUCTS
JOURNAL OF PROTEOME RESEARCH
JOURNAL OF THE AMERICAN CHEMICAL SOCIETY
LANGMUIR
MACROMOLECULES
MOLECULAR PHARMACEUTICS
NANO LETTERS
ORGANIC LETTERS
ORGANIC PROCESS RESEARCH & DEVELOPMENT
ORGANOMETALLICS

Supplementary Table 2. **Journals in Cell Biology.**

AMERICAN JOURNAL OF PHYSIOLOGY-CELL PHYSIOLOGY
AMERICAN JOURNAL OF RESPIRATORY CELL AND MOLECULAR BIOLOGY
AUTOPHAGY
BIOCHIMICA ET BIOPHYSICA ACTA-MOLECULAR CELL RESEARCH
CANCER CELL
CELL
CELL AND TISSUE RESEARCH
CELL CYCLE
CELL DEATH & DISEASE
CELL DEATH AND DIFFERENTIATION
CELL METABOLISM
CELL REPORTS
CELL RESEARCH
CELL STEM CELL
CELLULAR AND MOLECULAR LIFE SCIENCES
CELLULAR PHYSIOLOGY AND BIOCHEMISTRY
CELLULAR SIGNALLING
COLD SPRING HARBOR PERSPECTIVES IN BIOLOGY
CURRENT BIOLOGY
CURRENT OPINION IN CELL BIOLOGY
CURRENT OPINION IN STRUCTURAL BIOLOGY
DEVELOPMENTAL CELL
EMBO JOURNAL
EMBO REPORTS
EXPERIMENTAL CELL RESEARCH
FASEB JOURNAL
FEBS LETTERS
GENES & DEVELOPMENT
INTERNATIONAL JOURNAL OF BIOCHEMISTRY & CELL BIOLOGY
JOURNAL OF CELL BIOLOGY
JOURNAL OF CELL SCIENCE
JOURNAL OF CELLULAR AND MOLECULAR MEDICINE
JOURNAL OF CELLULAR BIOCHEMISTRY
JOURNAL OF CELLULAR PHYSIOLOGY

JOURNAL OF LEUKOCYTE BIOLOGY
JOURNAL OF MOLECULAR AND CELLULAR CARDIOLOGY
MOLECULAR AND CELLULAR BIOCHEMISTRY
MOLECULAR AND CELLULAR BIOLOGY
MOLECULAR AND CELLULAR ENDOCRINOLOGY
MOLECULAR BIOLOGY OF THE CELL
MOLECULAR CELL
NATURE CELL BIOLOGY
NATURE MEDICINE
NATURE REVIEWS MOLECULAR CELL BIOLOGY
NATURE STRUCTURAL & MOLECULAR BIOLOGY
ONCOGENE
PLANT AND CELL PHYSIOLOGY
PLANT CELL CIENCE SIGNALING
SCIENCE TRANSLATIONAL MEDICINE
STEM CELLS
STRUCTURE
TRENDS IN CELL BIOLOGY

Supplementary Table 3. **Journals in Neuroscience.**

ACTA NEUROPATHOLOGICA
ANNALS OF NEUROLOGY
BEHAVIOURAL BRAIN RESEARCH
BIOLOGICAL PSYCHIATRY
BRAIN
BRAIN BEHAVIOR AND IMMUNITY
BRAIN RESEARCH
CEREBRAL CORTEX
CLINICAL NEUROPHYSIOLOGY
CURRENT OPINION IN NEUROBIOLOGY
EUROPEAN JOURNAL OF NEUROLOGY
EUROPEAN JOURNAL OF NEUROSCIENCE
EXPERIMENTAL BRAIN RESEARCH
EXPERIMENTAL NEUROLOGY
FRONTIERS IN HUMAN NEUROSCIENCE
GAIT & POSTURE
GLIA
HUMAN BRAIN MAPPING
JOURNAL OF ALZHEIMERS DISEASE
JOURNAL OF CEREBRAL BLOOD FLOW AND METABOLISM
JOURNAL OF COGNITIVE NEUROSCIENCE
JOURNAL OF COMPARATIVE NEUROLOGY
JOURNAL OF NEUROCHEMISTRY
JOURNAL OF NEUROIMMUNOLOGY
JOURNAL OF NEUROPHYSIOLOGY
JOURNAL OF NEUROSCIENCE
JOURNAL OF NEUROSCIENCE METHODS
JOURNAL OF NEUROSCIENCE RESEARCH
JOURNAL OF NEUROTRAUMA
JOURNAL OF PHYSIOLOGY-LONDON
JOURNAL OF THE NEUROLOGICAL SCIENCES
MOLECULAR NEUROBIOLOGY
MOLECULAR PSYCHIATRY
MULTIPLE SCLEROSIS JOURNAL

MUSCLE & NERVE
NATURE NEUROSCIENCE
NEURAL COMPUTATION
NEURAL NETWORKS
NEUROBIOLOGY OF AGING
NEUROBIOLOGY OF DISEASE
NEUROIMAGE
NEURON
NEUROPHARMACOLOGY
NEUROPSYCHOLOGIA
NEUROPSYCHOPHARMACOLOGY
NEUROREPORT
NEUROSCIENCE
NEUROSCIENCE AND BIOBEHAVIORAL REVIEWS
NEUROSCIENCE LETTERS
PAIN
PHARMACOLOGY BIOCHEMISTRY AND BEHAVIOR
PROGRESS IN NEUROBIOLOGY
PSYCHONEUROENDOCRINOLOGY
PSYCHOPHARMACOLOGY
PSYCHOPHYSIOLOGY
SLEEP
TRENDS IN COGNITIVE SCIENCES
TRENDS IN NEUROSCIENCES
VISION RESEARCH

Supplementary Table 4. **Journals in Physics (published by American Physical Society).**

PHYSICAL REVIEW A
PHYSICAL REVIEW ACCELERATORS AND BEAMS
PHYSICAL REVIEW APPLIED
PHYSICAL REVIEW B
PHYSICAL REVIEW C
PHYSICAL REVIEW D
PHYSICAL REVIEW E
PHYSICAL REVIEW LETTERS
PHYSICAL REVIEW X

Supplementary Table 5. Values of the thresholds corresponding to the top 10% in terms of institutional prestige, publications and citations, for each year and each discipline.

First year	Discipline	Institutional prestige	Publications	Citations
1980	Cell Biology	51.83	5	19
1980	Chemistry	46.36	5	9
1980	Neuroscience	19.77	6	13
1980	Physics	30.75	6	13
1981	Cell Biology	36.91	5	14
1981	Chemistry	46.53	5	8
1981	Neuroscience	19.10	5	12
1981	Physics	31.55	6	13
1982	Cell Biology	33.55	4	14
1982	Chemistry	49.99	5	9
1982	Neuroscience	19.20	6	12
1982	Physics	31.93	5	13
1983	Cell Biology	47.69	5	17
1983	Chemistry	46.68	5	11
1983	Neuroscience	19.13	5	11
1983	Physics	33.57	5	17
1984	Cell Biology	37.60	4	17
1984	Chemistry	45.81	6	9
1984	Neuroscience	20.84	5	11
1984	Physics	34.13	6	17
1985	Cell Biology	37.52	4	18
1985	Chemistry	44.51	5	9
1985	Neuroscience	19.80	5	11
1985	Physics	32.26	6	16
1986	Cell Biology	32.76	5	19
1986	Chemistry	45.20	5	9
1986	Neuroscience	19.18	5	10
1986	Physics	29.85	5	13
1987	Cell Biology	35.91	5	23
1987	Chemistry	44.54	5	8
1987	Neuroscience	18.29	5	11
1987	Physics	31.23	5	17

1988	Cell Biology	37.26	4	21
1988	Chemistry	43.33	5	10
1988	Neuroscience	19.77	6	15
1988	Physics	31.16	6	15
1989	Cell Biology	36.32	4	25
1989	Chemistry	41.60	5	9
1989	Neuroscience	22.61	5	13
1989	Physics	31.07	5	13
1990	Cell Biology	37.09	4	19
1990	Chemistry	43.34	6	11
1990	Neuroscience	20.99	5	16
1990	Physics	28.27	5	13
1991	Cell Biology	37.51	4	25
1991	Chemistry	46.42	5	11
1991	Neuroscience	18.54	5	12
1991	Physics	30.44	5	14
1992	Cell Biology	36.45	4	26
1992	Chemistry	43.05	6	11
1992	Neuroscience	18.47	5	12
1992	Physics	26.60	6	16
1993	Cell Biology	33.60	4	21
1993	Chemistry	39.26	5	10
1993	Neuroscience	19.55	5	12
1993	Physics	29.73	6	15
1994	Cell Biology	35.96	4	16
1994	Chemistry	40.69	5	10
1994	Neuroscience	21.90	5	11
1994	Physics	26.53	6	15
1995	Cell Biology	35.87	4	20
1995	Chemistry	41.70	6	13
1995	Neuroscience	21.75	5	11
1995	Physics	28.61	6	20
1996	Cell Biology	31.31	4	21
1996	Chemistry	42.74	6	13
1996	Neuroscience	21.19	5	11

1996	Physics	28.58	7	20
1997	Cell Biology	30.08	5	38
1997	Chemistry	44.30	6	11
1997	Neuroscience	21.76	6	13
1997	Physics	26.90	7	20
1998	Cell Biology	30.42	6	42
1998	Chemistry	42.39	7	15
1998	Neuroscience	20.42	6	14
1998	Physics	27.29	8	22

Supplementary Table 6. **Binomial tests for matched pairs of junior researchers.** Junior researchers are matched based on the institutional prestige they are embedded in, the citations received and the number of papers published during their first 3 career years. One researcher per pair is either assigned to the treatment group (those who coauthored at least one paper with a top scientist). We compute the percentage of pairs in which the junior researcher who coauthored with a top scientist had a better performance with respect to the following quantities (statistical significance refers to a one-tailed binomial test): (A) citations received in career years 1-20; (B) citations received in career years 4-20 excluding those received by the papers published in the first 3 career years; (C) citations received per paper published during career years 4-20; (D) number of different top scientists (per paper published) with whom the researcher has coauthored papers in career years 4-20 (excluding those already accounted in the first 3 career years for the treatment group); (E) number of times (per paper published) the researcher has coauthored papers with a top scientist in career years from 4 to 20 (excluding those already accounted in the first 3 career years for the treatment group). (*: $p < 0.05$; **: $p < 0.01$; ***: $p < 0.001$; NS: not significant). Numbers in brackets denote standard errors.

	Cell Biology			Chemistry			Neuroscience			Physics		
No. authors	2,324			5,635			5,605			5,414		
No. pairs	468			1,443			1,602			1,362		
	Top co.	Control	p	Top co.	Control	p	Top co.	Control	p	Top co.	Control	p
Inst. prestige	22.40	22.24	NS	26.85	26.99	NS	10.52	10.66	NS	19.58	19.69	NS
	(0.57)	(0.66)		(0.32)	(0.39)		(0.17)	(0.19)		(0.24)	(0.27)	
Productivity	2.36	2.37	NS	3.09	3.13	NS	3.13	3.09	NS	3.35	3.25	NS
	(0.06)	(0.06)		(0.04)	(0.05)		(0.04)	(0.04)		(0.05)	(0.05)	
Cit. (years 1-3)	4.80	4.71	NS	4.06	3.95	NS	3.95	3.92	NS	4.22	4.19	NS
	(0.15)	(0.14)		(0.08)	(0.08)		(0.07)	(0.07)		(0.08)	(0.08)	
(A)	57.5%		**	56.3%		***	58.4%		***	61.2%		***
(B)	56.6%		**	56.8%		***	57.9%		**	61.4%		***
(C)	48.1%		NS	52.3%		0.08	48.4%		NS	56.0%		***
(D)	50.6%		NS	57.2%		***	52.9%		*	56.8%		***
(E)	52.6%		NS	59.7%		***	54.7%		***	59.2%		***

Supplementary Table 7. **Matched pair analysis performed over the first 5 career years.** Junior researchers are matched based on the institution prestige they are embedded in, their productivity (measured by the number of papers published) and the number of citations received during their first 5 career years. We only consider authors who have no more than 15 citations in the first 5 years. Then one researcher per pair is either assigned to the treatment group (those who coauthored at least one paper with a top scientist) or the control group, and we compute the average of the following quantities across the two groups: (A) Citations received in career years 1-20. (B) Citations received in career years 6-20 excluding those received by the papers published in the first 5 career years. (C) Citations received per paper published during career years 6-20. (D) Number of different top scientists (per paper published) with whom the researcher has coauthored papers in career years 6-20 (excluding those already accounted in the first 5 career years for the treatment group). (E) Number of times (per paper published) the researcher has coauthored papers with a top scientist in career years from 6 to 20 (excluding those already accounted in the first 5 career years for the treatment group). (*: $p < 0.05$; **: $p < 0.01$; ***: $p < 0.001$; NS: not significant). Numbers in brackets denote standard errors.

	Cell Biology			Chemistry			Neuroscience			Physics		
No. authors	$1,796$			$4,631$			$4,259$			$4,083$		
No. pairs	400			$1,359$			$1,365$			$1,177$		
	Treat	Control	p	Treat	Control	p	Treat	Control	p	Treat	Control	p
Inst. prestige	19.02	18.88	NS	25.80	25.40	NS	9.81	10.00	NS	18.26	18.16	NS
	(0.55)	(0.67)		(0.31)	(0.38)		(0.17)	(0.19)		(0.23)	(0.26)	
Productivity	3.35	3.48	NS	4.36	4.44	NS	4.39	4.30	NS	4.72	4.64	NS
	(0.07)	(0.09)		(0.06)	(0.07)		(0.06)	(0.06)		(0.06)	(0.07)	
Cit. (years 1-5)	7.97	8.00	NS	7.35	7.33	NS	7.51	7.40	NS	7.46	7.44	NS
	(0.22)	(0.23)		(0.11)	(0.12)		(0.12)	(0.12)		(0.13)	(0.13)	
(A)	251.35	188.56	**	243.23	193.66	***	278.78	234.09	***	286.08	208.53	***
	(14.30)	(13.44)		(7.36)	(5.88)		(8.50)	(7.42)		(9.60)	(6.33)	
(B)	230.71	168.09	**	220.18	169.80	***	248.94	203.66	***	264.33	187.58	***
	(14.19)	(13.24)		(7.31)	(5.76)		(8.28)	(7.10)		(9.51)	(6.25)	
(C)	11.80	8.82	***	7.07	6.00	***	10.54	8.61	***	8.93	7.44	***
	(0.43)	(0.37)		(0.12)	(0.11)		(0.21)	(0.17)		(0.22)	(0.20)	
(D)	2.85	1.98	***	4.01	2.31	***	2.80	2.03	***	3.33	1.83	***
	(0.16)	(0.14)		(0.12)	(0.08)		(0.08)	(0.07)		(0.13)	(0.08)	
(E)	4.07	2.69	***	8.00	4.64	***	5.27	3.75	***	7.02	3.91	***
	(0.30)	(0.21)		(0.32)	(0.22)		(0.22)	(0.19)		(0.35)	(0.20)	

Supplementary Table 8. **Matched pair analysis with number of coauthors as additional covariate.** The table reports the results of the matched pair analysis discussed in the main text extended to include the number of unique coauthors that junior researchers had during their first 3 career years. Junior researchers are matched based on this number, and on the institutional prestige they are embedded in, their productivity (measured by the number of papers published), the number of citations received, and the number of unique coauthors they have during their first 3 career years. We consider authors with no more than 10 citations and no more than 10 unique coauthors in their first 3 career years. One researcher per pair is either assigned to the treatment group (those who coauthored at least one paper with a top scientist) or the control group, and we compute the average of the following quantities across the two groups: (A) Citations received in career years 1-20. (B) Citations received in career years 4-20 excluding those received by the papers published in the first 3 career years. (C) Citations received per paper published during career years 4-20. (D) Number of different top scientists (per paper published) with whom the researcher has coauthored papers in career years 4-20 (excluding those already accounted in the first 3 career years for the treatment group). (E) Number of times (per paper published) the researcher has coauthored papers with a top scientist in career years from 4 to 20 (excluding those already accounted in the first 3 career years for the treatment group). (*: $p < 0.05$; **: $p < 0.01$; ***: $p < 0.001$; NS: not significant). Numbers in brackets denote standard errors.

	Cell Biology			Chemistry			Neuroscience			Physics		
No. authors	$2,280$			$5,437$			$5,343$			$5,179$		
No. pairs	449			$1,317$			$1,429$			$1,203$		
	Treat	Control	p	Treat	Control	p	Treat	Control	p	Treat	Control	p
Inst. prestige	22.63	21.98	NS	27.11	27.13	NS	10.67	11.05	NS	19.68	19.64	NS
	(0.59)	(0.69)		(0.34)	(0.40)		(0.19)	(0.21)		(0.26)	(0.28)	
Productivity	2.26	2.25	NS	2.92	2.83	NS	2.89	2.87	NS	3.11	3.06	NS
	(0.05)	(0.06)		(0.04)	(0.04)		(0.04)	(0.04)		(0.05)	(0.05)	
Cit. (years 1-3)	4.74	4.40	NS	3.95	3.64	**	3.79	3.67	NS	4.06	3.97	NS
	(0.15)	(0.14)		(0.08)	(0.08)		(0.07)	(0.08)		(0.09)	(0.09)	
Coauthors	4.20	4.15	NS	4.84	4.53	***	4.70	4.44	***	4.53	4.36	NS
	(0.11)	(0.11)		(0.07)	(0.06)		(0.06)	(0.06)		(0.07)	(0.07)	
(A)	358.32	281.82	**	296.12	253.29	***	364.22	332.40	*	392.09	294.68	***
	(19.01)	(16.00)		(8.88)	(7.72)		(10.57)	(12.23)		(13.60)	(10.69)	
(B)	313.79	246.41	**	256.47	217.37	***	302.10	281.35	NS	343.33	257.49	***
	(19.01)	(15.38)		(8.59)	(7.33)		(9.76)	(11.12)		(12.66)	(10.24)	
(C)	14.58	11.37	***	7.59	6.83	***	12.15	10.33	***	10.24	8.66	***
	(0.49)	(0.46)		(0.13)	(0.12)		(0.25)	(0.22)		(0.23)	(0.23)	
(D)	3.55	2.87	**	4.74	3.37	***	3.35	2.77	***	4.04	3.00	***
	(0.17)	(0.16)		(0.13)	(0.11)		(0.09)	(0.09)		(0.14)	(0.12)	
(E)	5.24	4.22	*	10.43	7.09	***	6.70	5.67	**	9.38	6.64	***
	(0.30)	(0.28)		(0.39)	(0.31)		(0.25)	(0.27)		(0.40)	(0.35)	

Supplementary Figures

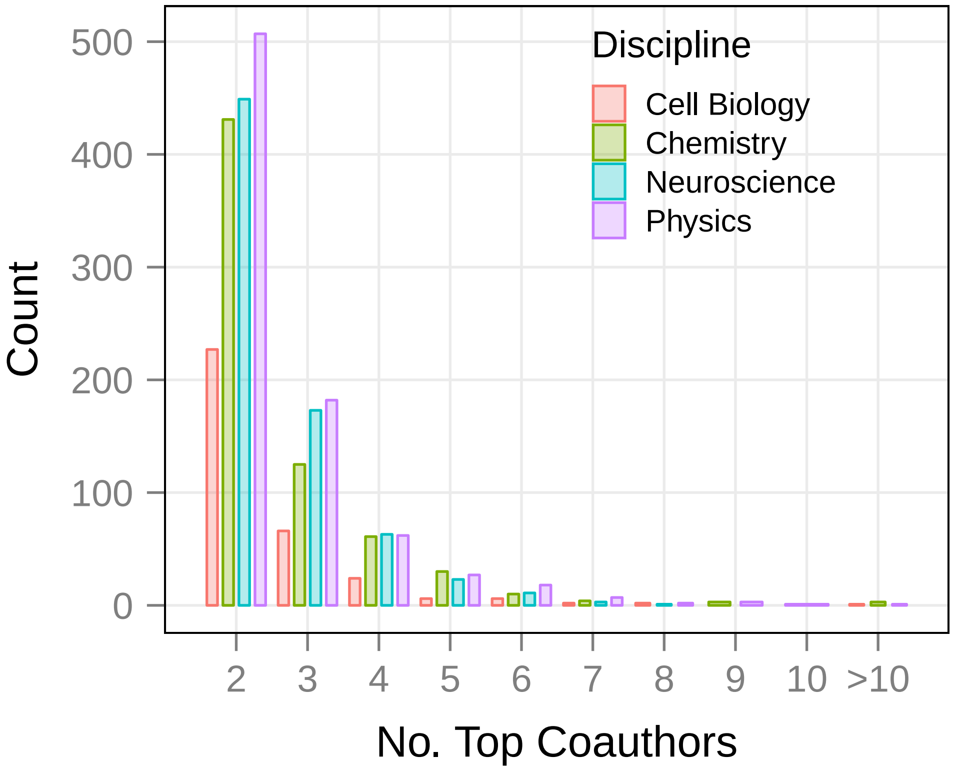

Supplementary Figure 1. **Distribution of junior researchers with more than one top coauthor.** The histograms report - for each discipline - the number of junior researchers in our dataset who coauthored work in their first 3 career years with more than one top scientist.

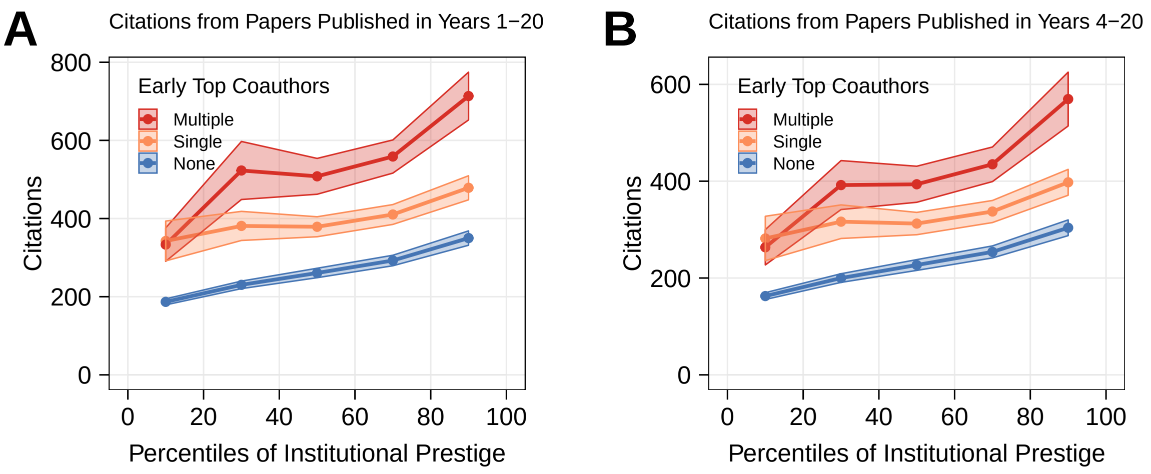

Supplementary Figure 2. **Relationship between early career institutional prestige and long-term impact.** **A** Total citations received in the first 20 career years. **B** Total citations received in career years 4-20. In both panels authors are grouped based on whether in their first 3 career years they coauthored papers with one (orange), multiple (red) or no (blue) top scientists.

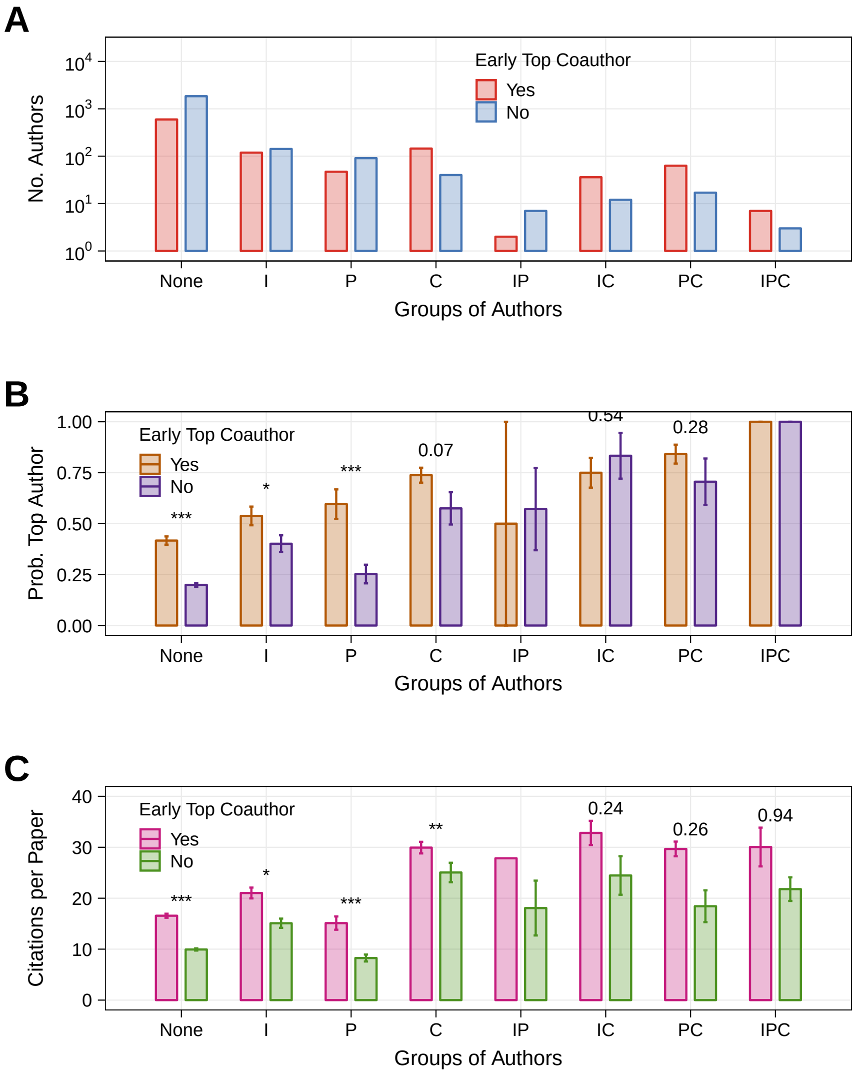

Supplementary Figure 3. **Relationship between long term impact and early career performance in Cell Biology.** **A** Number of junior researchers belonging to the top 10% in various categories of early career performance (I denotes institutional prestige, P denotes productivity, C denotes citations received. All three such quantities are computed based on the first 3 career years). **B** Probability for authors belonging to each group of being a top scientist in their 20th career year. **C** Number of citations received per paper published by authors belonging to each group between their 4th and 20th career year. In all panels, we report the p-values obtained via t-tests to assess the statistical significance of differences between the sub-group of junior researchers who coauthor work with a top scientist in the first 3 career years and the sub-group of those who do not. *: $p < 0.05$; **: $p < 0.01$; ***: $p < 0.001$.

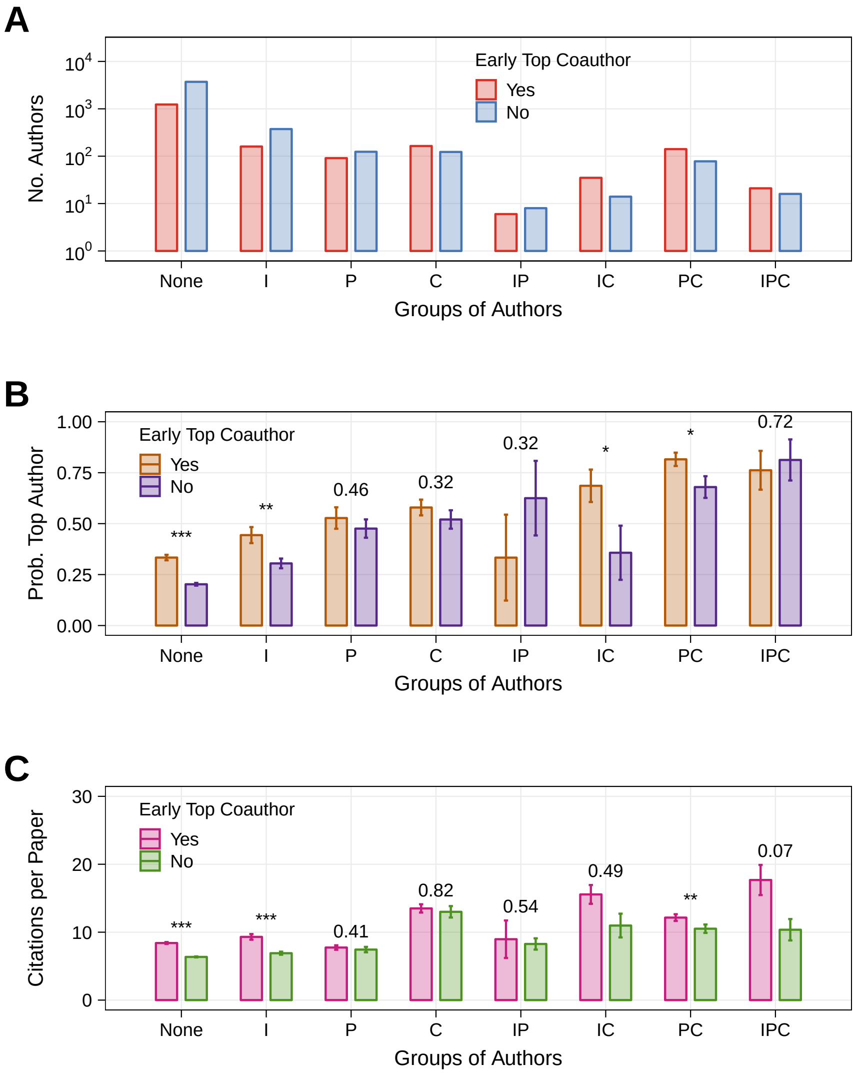

Supplementary Figure 4. **Relationship between long term impact and early career performance in Chemistry. A** Number of junior researchers belonging to the top 10% in various categories of early career performance (I denotes institutional prestige, P denotes productivity, C denotes citations received. All three such quantities are computed based on the first 3 career years). **B** Probability for authors belonging to each group of being a top scientist in their 20th career year. **C** Number of citations received per paper published by authors belonging to each group between their 4th and 20th career year. In all panels, we report the p-values obtained via t-tests to assess the statistical significance of differences between the sub-group of junior researchers who coauthor work with a top scientist in the first 3 career years and the sub-group of those who do not. *: $p < 0.05$; **: $p < 0.01$; ***: $p < 0.001$.

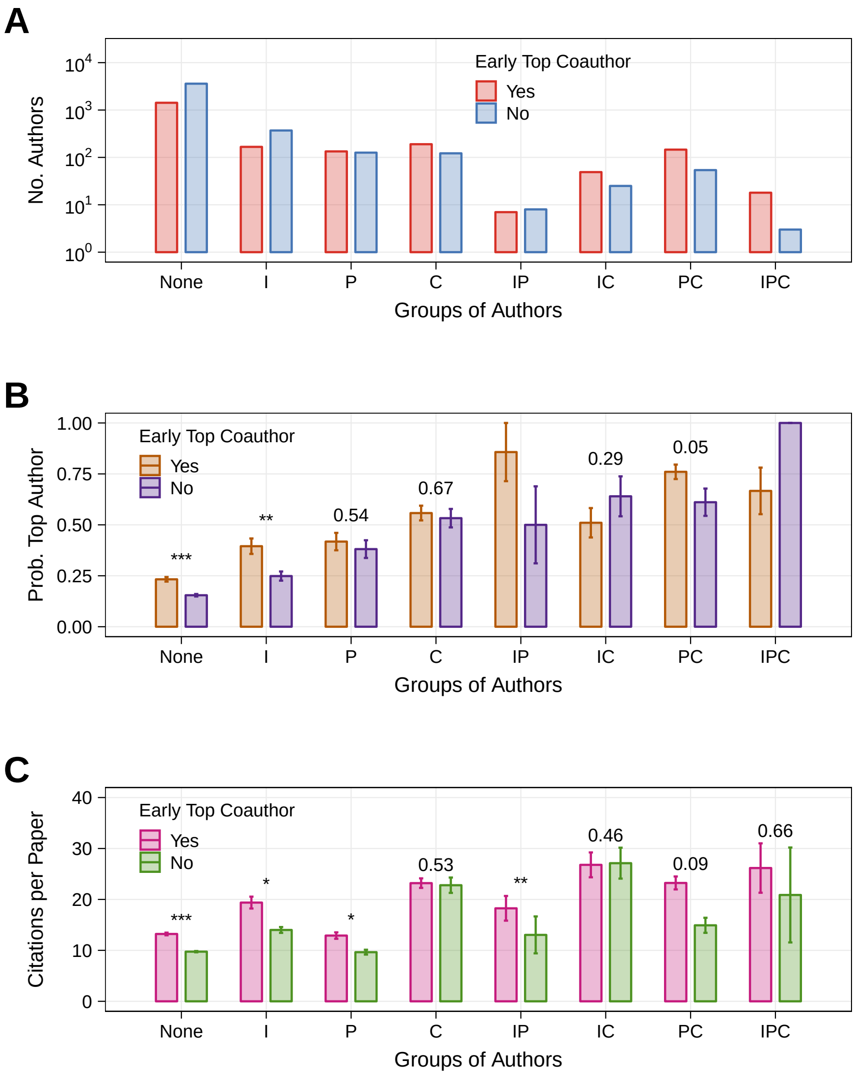

Supplementary Figure 5. **Relationship between long term impact and early career performance in Neuroscience. A** Number of junior researchers belonging to the top 10% in various categories of early career performance (I denotes institutional prestige, P denotes productivity, C denotes citations received. All three such quantities are computed based on the first 3 career years). **B** Probability for authors belonging to each group of being a top scientist in their 20th career year. **C** Number of citations received per paper published by authors belonging to each group between their 4th and 20th career year. In all panels, we report the p-values obtained via t-tests to assess the statistical significance of differences between the sub-group of junior researchers who coauthor work with a top scientist in the first 3 career years and the sub-group of those who do not. *: $p < 0.05$; **: $p < 0.01$; ***: $p < 0.001$.

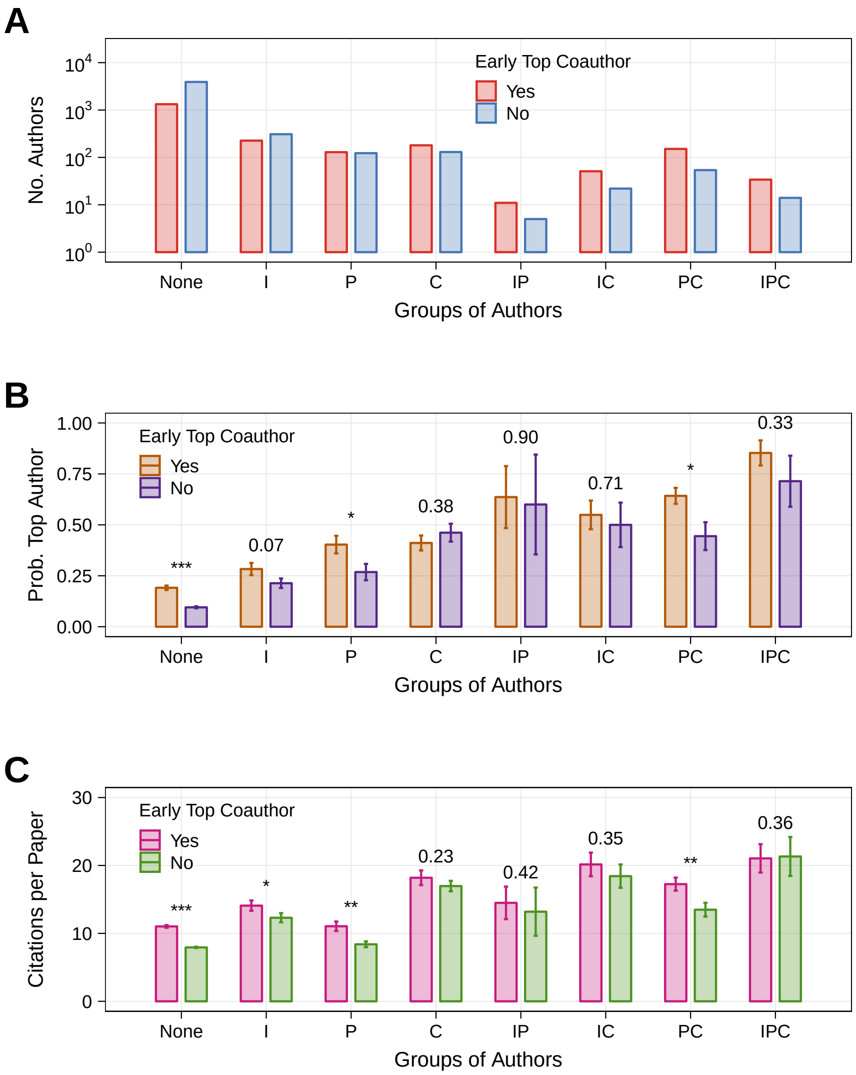

Supplementary Figure 6. **Relationship between long term impact and early career performance in Physics. A** Number of junior researchers belonging to the top 10% in various categories of early career performance (I denotes institutional prestige, P denotes productivity, C denotes citations received. All three such quantities are computed based on the first 3 career years). **B** Probability for authors belonging to each group of being a top scientist in their 20th career year. **C** Number of citations received per paper published by authors belonging to each group between their 4th and 20th career year. In all panels, we report the p-values obtained via t-tests to assess the statistical significance of differences between the sub-group of junior researchers who coauthor work with a top scientist in the first 3 career years and the sub-group of those who do not. *: $p < 0.05$; **: $p < 0.01$; ***: $p < 0.001$.

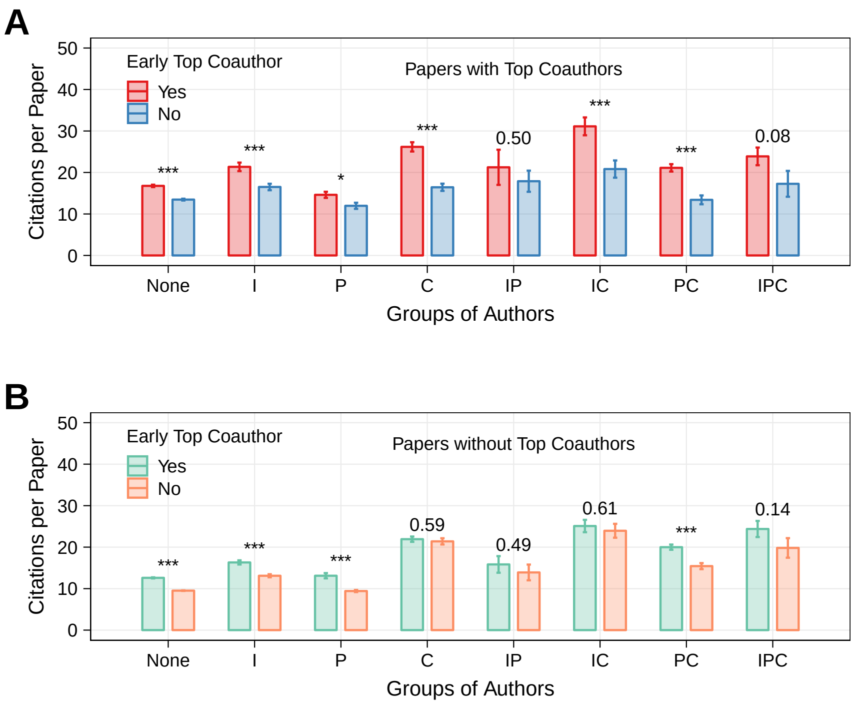

Supplementary Figure 7. **Relationship between early career performance and long term impact with / without citations obtained with top coauthors.** The two panels show the number of citations received per paper published in career years 4-20 by junior researchers belonging to the top 10% of their field in various categories (I denotes institutional prestige, P denotes productivity, C denotes citations received. All three such quantities are computed based on the first 3 career years). Panel **A** shows the citations obtained only when coauthoring papers with top scientists, panel **B** shows the citations obtained when publishing papers without top scientists as coauthors. *: $p < 0.05$; **: $p < 0.01$; ***: $p < 0.001$.

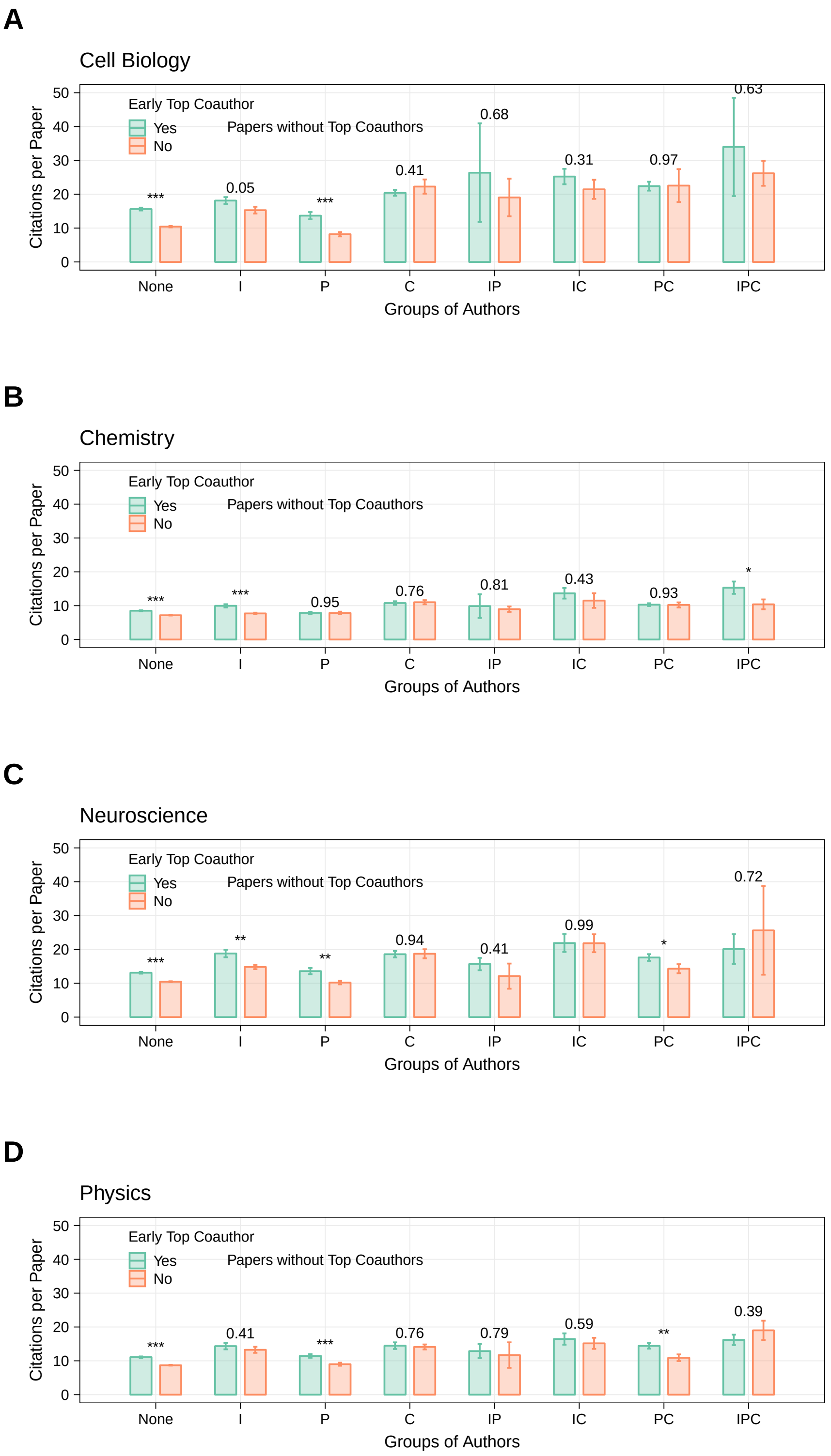

Supplementary Figure 8. **Relationship between early career performance and long term impact with / without citations obtained with top coauthors in each discipline.** The four panels show the number of citations received per paper published in career years 4-20 by junior researchers belonging to the top 10% of their discipline in various categories (I denotes institutional prestige, P denotes productivity, C denotes citations received. All three such quantities are computed based on the first 3 career years) when publishing papers without top scientists as coauthors. Panel **A** shows results for Cell Biology, panel **B** for Chemistry, panel **C** for Neuroscience, panel **D** for Physics, respectively. *: $p < 0.05$; **: $p < 0.01$; ***: $p < 0.001$.

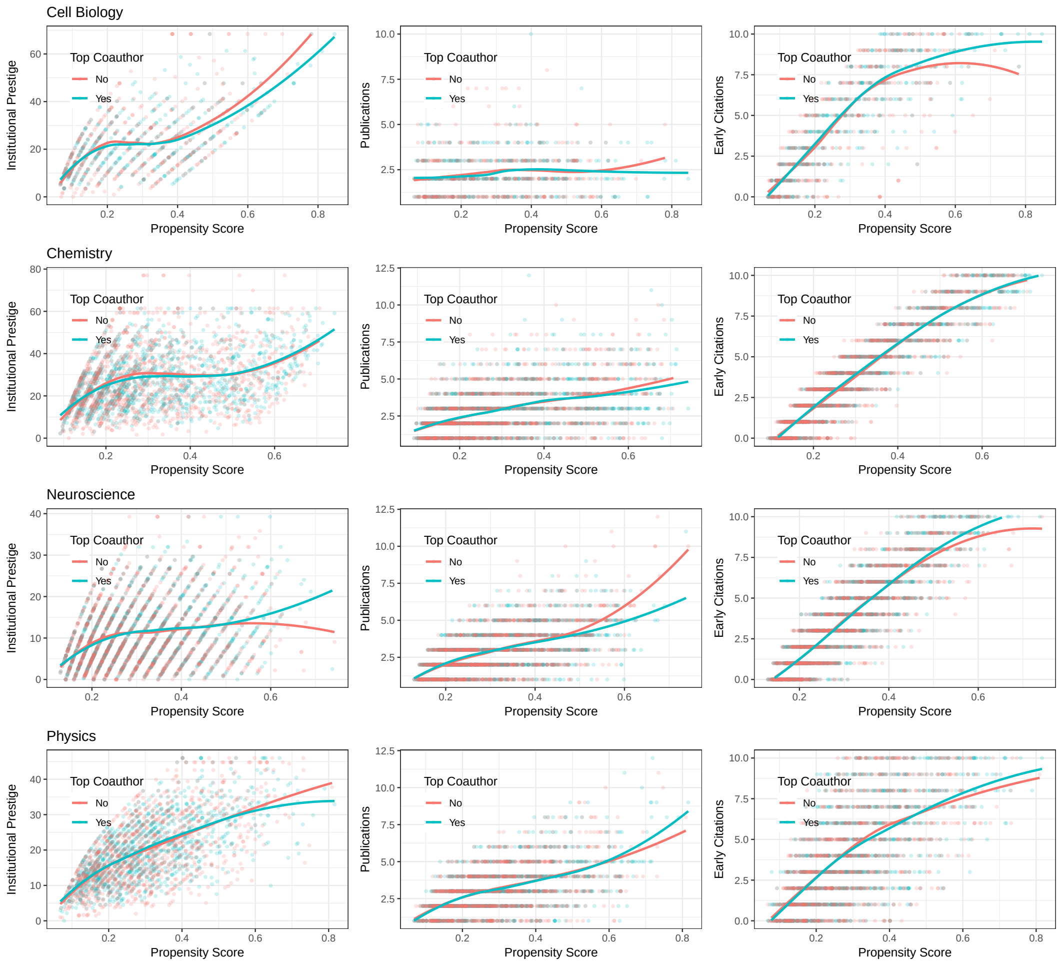

Supplementary Figure 9. **Propensity score plots for matched-pair analysis.** Each plot shows the propensity scores for each junior researchers in our matched pair analysis (see Table 1 in the main text), for all disciplines and all covariates (i.e., institutional prestige, number of publications, and number of citations). Values for junior researchers who have (do not have) coauthored at least one paper with a top scientist are shown in green (red). Solid lines show the average values of all covariates as functions of the propensity scores.
