# Supplementary material for: Early coauthorship with top scientists predicts success in academic careers
Source: Nat Commun. 2019 Nov 15;10:5170. doi: 10.1038/s41467-019-13130-4 (PMC6858367; doi:10.1038/s41467-019-13130-4)
Supplement: Supplementary file 3 — Reporting Summary [file 41467_2019_13130_MOESM3_ESM.pdf]

nature research

Corresponding author(s): Giacomo Livan

Last updated by author(s): Oct 11, 2019

Reporting Summary

Nature Research wishes to improve the reproducibility of the work that we publish. This form provides structure for consistency and transparency in reporting. For further information on Nature Research policies, see Authors & Referees and the Editorial Policy Checklist.

Statistics

For all statistical analyses, confirm that the following items are present in the figure legend, table legend, main text, or Methods section.

n/a	Confirmed	
☐	☒	The exact sample size (*n*) for each experimental group/condition, given as a discrete number and unit of measurement
☐	☒	A statement on whether measurements were taken from distinct samples or whether the same sample was measured repeatedly
☐	☒	The statistical test(s) used AND whether they are one- or two-sided<br>*Only common tests should be described solely by name; describe more complex techniques in the Methods section.*
☐	☒	A description of all covariates tested
☒	☐	A description of any assumptions or corrections, such as tests of normality and adjustment for multiple comparisons
☐	☒	A full description of the statistical parameters including central tendency (e.g. means) or other basic estimates (e.g. regression coefficient) AND variation (e.g. standard deviation) or associated estimates of uncertainty (e.g. confidence intervals)
☐	☒	For null hypothesis testing, the test statistic (e.g. *F*, *t*, *r*) with confidence intervals, effect sizes, degrees of freedom and *P* value noted<br>*Give P values as exact values whenever suitable.*
☒	☐	For Bayesian analysis, information on the choice of priors and Markov chain Monte Carlo settings
☒	☐	For hierarchical and complex designs, identification of the appropriate level for tests and full reporting of outcomes
☒	☐	Estimates of effect sizes (e.g. Cohen's *d*, Pearson's *r*), indicating how they were calculated

Our web collection on statistics for biologists contains articles on many of the points above.

Software and code

Policy information about availability of computer code

Data collection: No specific software was used to collect data

Data analysis: The code for used to perform pair matching is available at https://cran.r-project.org/web/packages/MatchIt/index.html

For manuscripts utilizing custom algorithms or software that are central to the research but not yet described in published literature, software must be made available to editors/reviewers. We strongly encourage code deposition in a community repository (e.g. GitHub). See the Nature Research guidelines for submitting code & software for further information.

Data

Policy information about availability of data

All manuscripts must include a data availability statement. This statement should provide the following information, where applicable:

- Accession codes, unique identifiers, or web links for publicly available datasets
- A list of figures that have associated raw data
- A description of any restrictions on data availability

The APS data used in the paper are publicly accessible and can be downloaded via journals.aps.org/datasets. The other publication and citation data are available via Web of Science https://wok.mimas.ac.uk/

Field-specific reporting

Please select the one below that is the best fit for your research. If you are not sure, read the appropriate sections before making your selection.

☐ Life sciences ☒ Behavioural & social sciences ☐ Ecological, evolutionary & environmental sciences

For a reference copy of the document with all sections, see nature.com/documents/nr-reporting-summary-flat.pdf

Behavioural & social sciences study design

All studies must disclose on these points even when the disclosure is negative.

Study description	The study quantifies the impact of early career coauthorship with top-cited scientistis on the long-term prospects of academic careers. This is done by quantitatively analyzing academic publication and citation data.
Research sample	The study relies on existing datasets. The APS data used in the paper are publicly accessible and can be downloaded via journals.aps.org/datasets. The other publication and citation data are available via Web of Science https://wok.mimas.ac.uk/
Sampling strategy	The sample sizes in all disciplines considered in the study were determined as follows. Publication and citation data were retained only for authors whose academic career lasted at least 20 years and whose papers received at least 10 citations from other works published in the journals considered in the study.
Data collection	The APS data were downloaded from journals.aps.org/datasets, while the datasets for the remaining disciplines were manually downloaded and compiled from https://wok.mimas.ac.uk/
Timing	The data were downloaded from the aforementioned sources in July 2018.
Data exclusions	As mentioned above, scientists whose career lasted less than 20 years or whose work received less than 10 citations were removed from the study.
Non-participation	n/a
Randomization	n/a

Reporting for specific materials, systems and methods

We require information from authors about some types of materials, experimental systems and methods used in many studies. Here, indicate whether each material, system or method listed is relevant to your study. If you are not sure if a list item applies to your research, read the appropriate section before selecting a response.

Materials & experimental systems

n/a	Involved in the study	
☒	☐	Antibodies
☒	☐	Eukaryotic cell lines
☒	☐	Palaeontology
☒	☐	Animals and other organisms
☒	☐	Human research participants
☒	☐	Clinical data

Methods

n/a	Involved in the study	
☒	☐	ChIP-seq
☒	☐	Flow cytometry
☒	☐	MRI-based neuroimaging
